# Supplementary material for: Thermal Decomposition Study on Li2O2 for Li2NiO2 Synthesis as a Sacrificing Positive Additive of Lithium-Ion Batteries
Source: Molecules. 2019 Dec 17;24(24):4624. doi: 10.3390/molecules24244624 (PMC6943730; doi:10.3390/molecules24244624)
Supplement: Supplementary file 1 [file molecules-24-04624-s001.pdf]

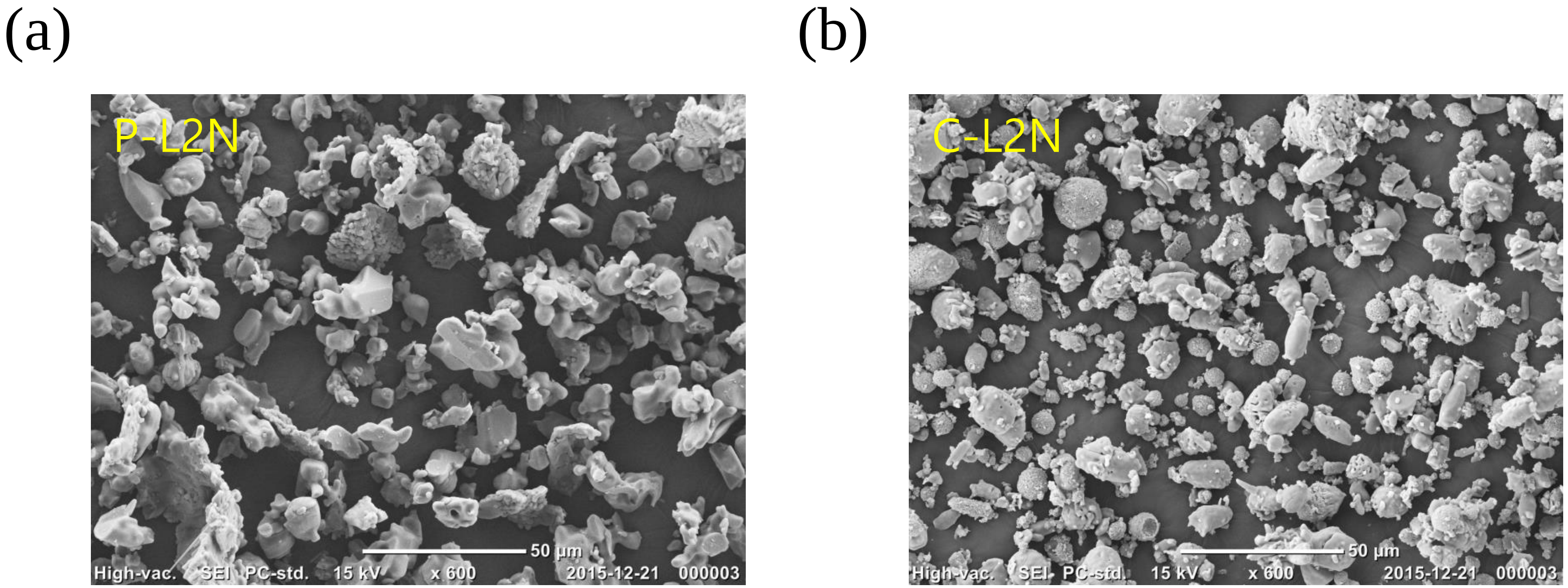

Figure. S1 SEM images of (a) P-L2N and (b) C-L2N

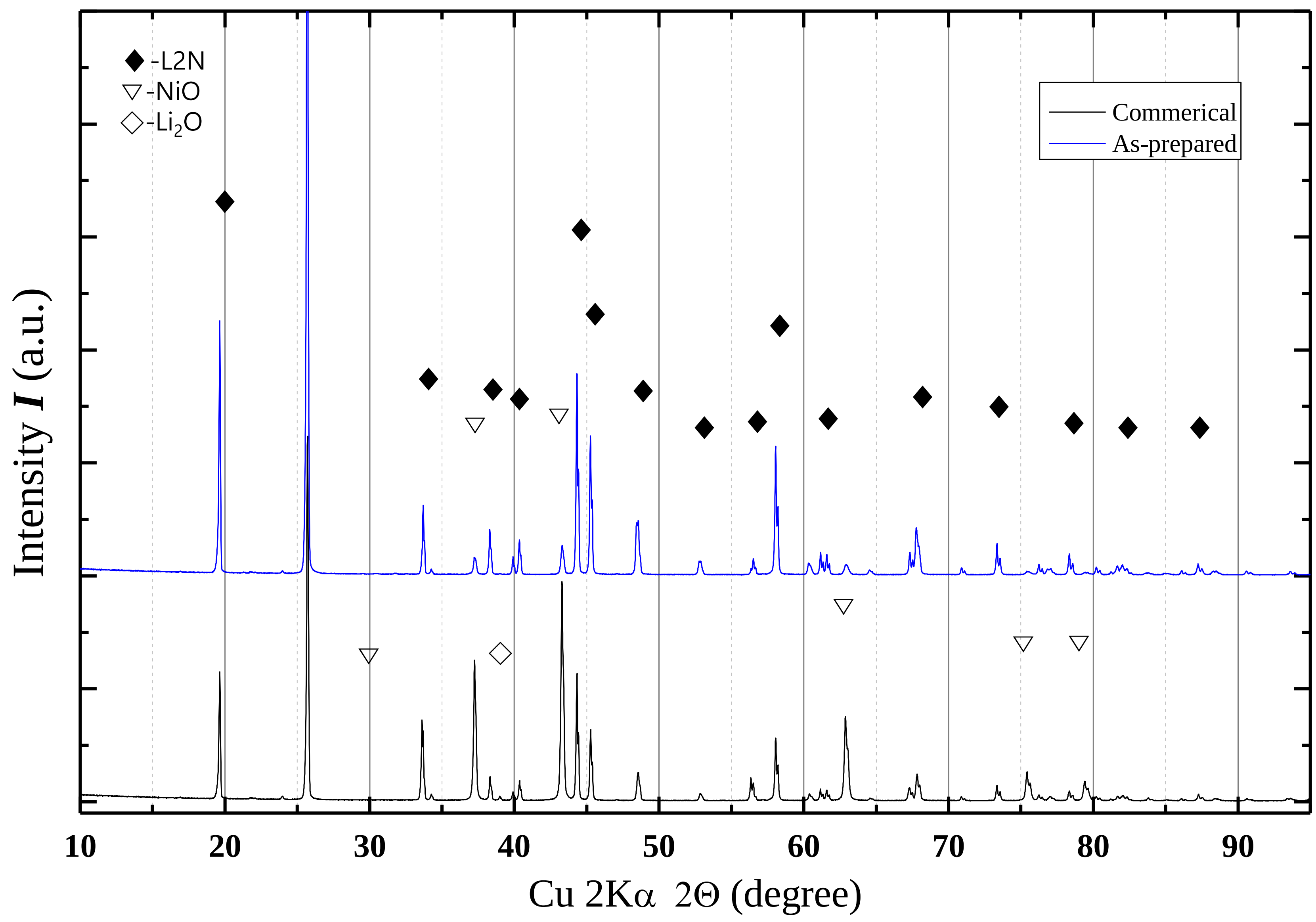

Figure. S2 XRD patterns of C-L2N (black) and P-L2N (blue)

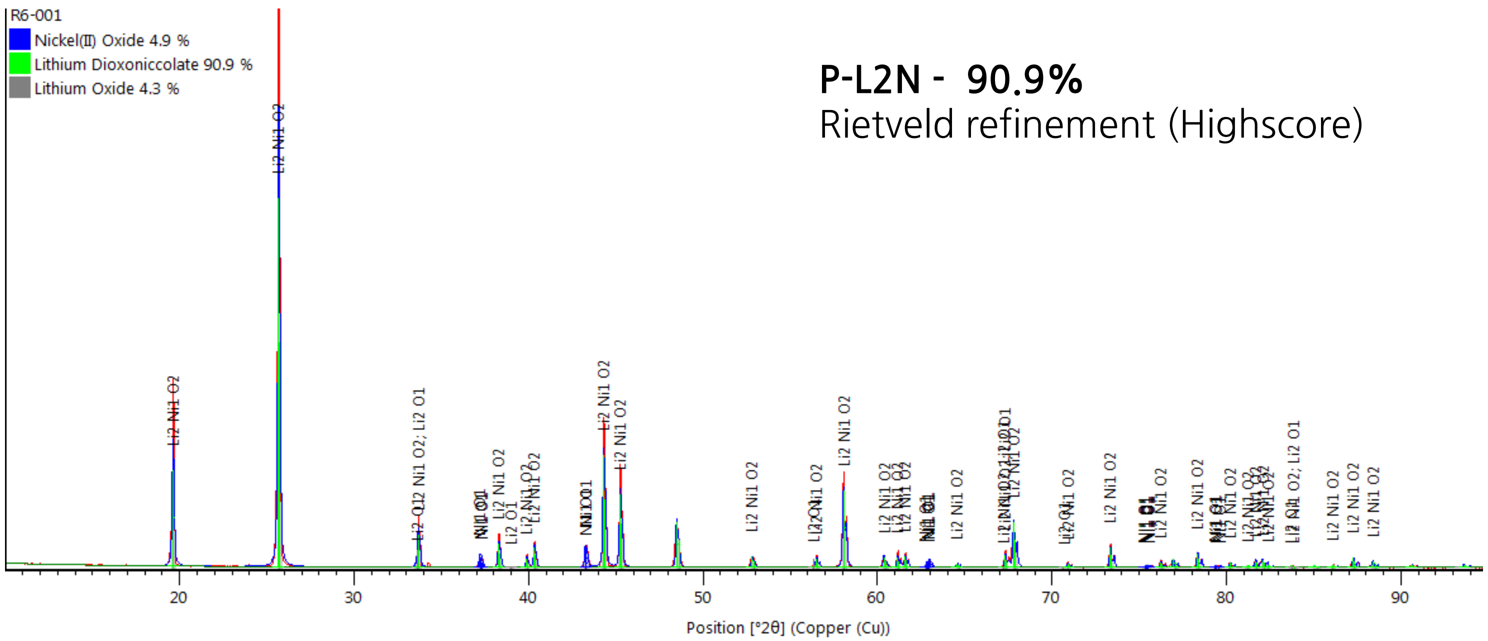

Figure. S3 Quantitative analysis result of P-L2N by Rietveld refinement

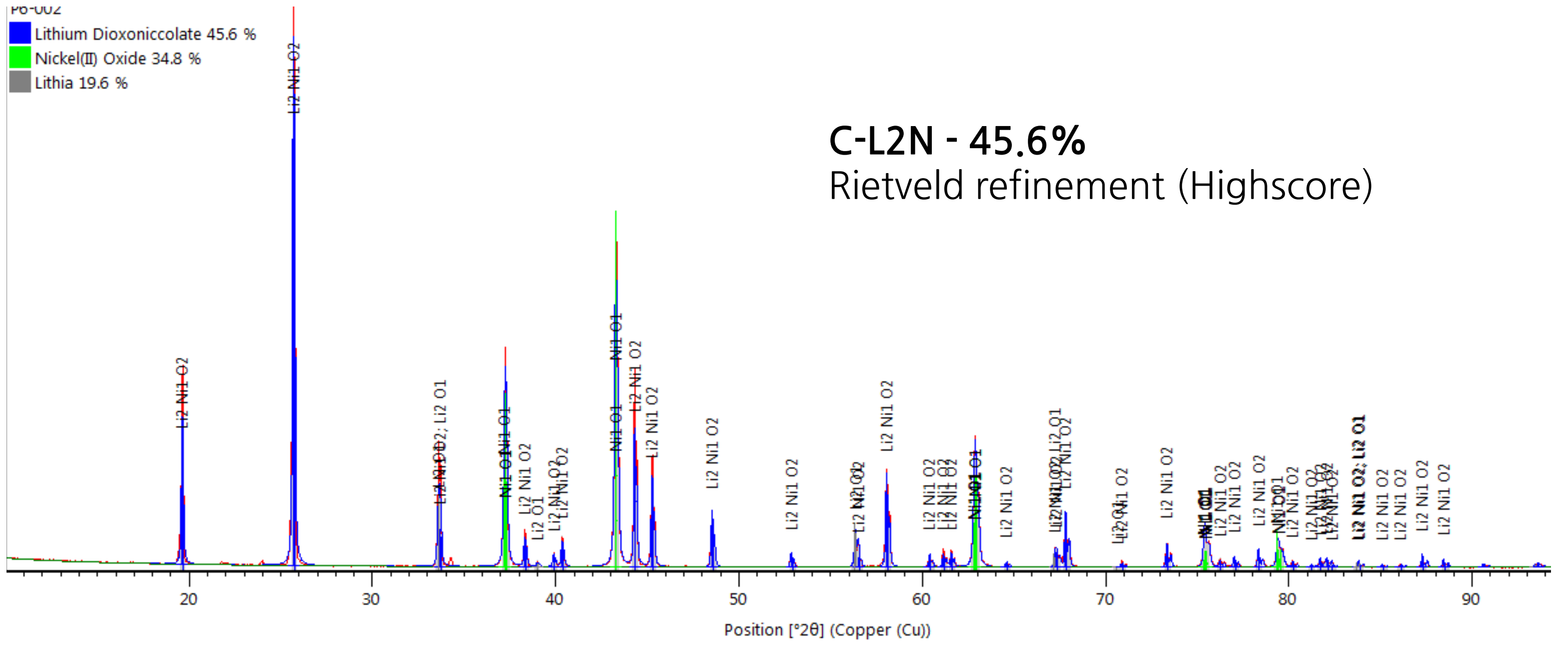

Figure. S4 Quantitative analysis result of C-L2N by Rietveld refinement
